# TsrA Regulates Virulence and Intestinal Colonization in *Vibrio cholerae*

**DOI:** 10.1128/mSphere.01014-20

**Published:** 2020-12-09

**Authors:** Cory D. DuPai, Ashley L. Cunningham, Aaron R. Conrado, Claus O. Wilke, Bryan W. Davies

**Affiliations:** a Department of Molecular Biosciences, University of Texas at Austin, Austin, Texas, USA; b Department of Integrative Biology, University of Texas at Austin, Austin, Texas, USA; c Center for Systems and Synthetic Biology, John Ring LaMontagne Center for Infectious Diseases, Institute for Cellular and Molecular Biology, University of Texas at Austin, Austin, Texas, USA; University of Kentucky

**Keywords:** *Vibrio cholerae*, gene regulation, H-NS, TsrA, computational biology, genetics, virulence regulation

## Abstract

Pathogenic strains of Vibrio cholerae require careful regulation of horizontally acquired virulence factors that are largely located on horizontally acquired genomic islands (HAIs). While TsrA, a *Vibrionaceae*-specific protein, is known to regulate the critical HAI virulence genes *toxT* and *ctxA*, its broader function throughout the genome is unknown. Here, we find that deletion of *tsrA* results in genomewide expression patterns that heavily correlate with those seen upon deletion of *hns*, a widely conserved bacterial protein that regulates V. cholerae virulence. This correlation is particularly strong for loci on HAIs, where all differentially expressed loci in the *ΔtsrA* mutant are also differentially expressed in the *Δhns* mutant. Correlation between TsrA and H-NS function extends to *in vivo* virulence phenotypes where deletion of *tsrA* compensates for the loss of ToxR activity in V. cholerae and promotes wild-type levels of mouse intestinal colonization. All in all, we find that TsrA broadly controls V. cholerae infectivity via repression of key HAI virulence genes and many other targets in the H-NS regulon.

**IMPORTANCE** Cholera is a potentially lethal disease that is endemic in much of the developing world. Vibrio cholerae, the bacterium underlying the disease, infects humans utilizing proteins encoded on horizontally acquired genetic material. Here, we provide evidence that TsrA, a *Vibrionaceae*-specific protein, plays a critical role in regulating these genetic elements and is essential for V. cholerae virulence in a mouse intestinal model.

## Abstract (precis)

Cholera is a potentially lethal disease that is endemic in much of the developing world. Vibrio cholerae, the bacterium underlying the disease, infects humans utilizing proteins encoded on horizontally acquired genetic material. Here, we provide evidence that TsrA, a *Vibrionaceae*-specific protein, plays a critical role in regulating these genetic elements and is essential for V. cholerae virulence in a mouse intestinal model.

## INTRODUCTION

Vibrio cholerae is the causative agent of the potentially lethal disease cholera. Several factors on the progenitor genome and horizontally acquired genetic islands (HAIs) (1–4) act in concert to control V. cholerae virulence gene expression. While multiple HAIs play some role in virulence (2, 5–11), genes on V. cholerae pathogenicity island 1 (VPI-1) and the cholera toxin (CTX) prophage are most involved with the major virulence pathway, the ToxR regulon (4, 12). ToxR and the histone-like nucleoid structuring protein (H-NS) coordinate to activate or repress, respectively, the master virulence regulator ToxT and other virulence genes on VPI-1 (4–7, 13–15). Since H-NS is highly abundant and broadly controls genomic structure and expression patterns (12, 16–26), factors that modulate and fine-tune H-NS repression are common in enteric bacteria (27–32). To date, however, no such factors have emerged in V. cholerae.

TsrA is a *Vibrionaceae*-specific protein that is by far most common in the genomes of organisms within the *Vibrio* genus, as determined via BLAST-based (33) protein homology searches (data not shown). TsrA has been shown to regulate type VI secretion system (T6SS) genes (namely, Hcp) in coordination with quorum-sensing pathways and further affects both expression of *toxT* and the ability of V. cholerae to colonize the small intestine (34). Despite this knowledge, TsrA’s larger impacts on V. cholerae gene regulation have not been explored. Here, we elaborate on previous findings and provide more clarity regarding TsrA’s impact on gene regulation throughout the V. cholerae genome. Our transcriptomics analyses demonstrate that TsrA mimics the ability of H-NS to repress acquired genetic elements on canonical pathogenicity islands. We further show that this gene plays a critical role in controlling intestinal colonization, with deletion of *tsrA* completely compensating for the attenuation observed when ToxR, an essential virulence gene regulator, is also deleted in an infant mouse intestinal model. Our findings illustrate a large role in controlling V. cholerae virulence for this small protein.

## RESULTS

### TsrA deletion promotes expression of H-NS regulon.

**FIG 1:**
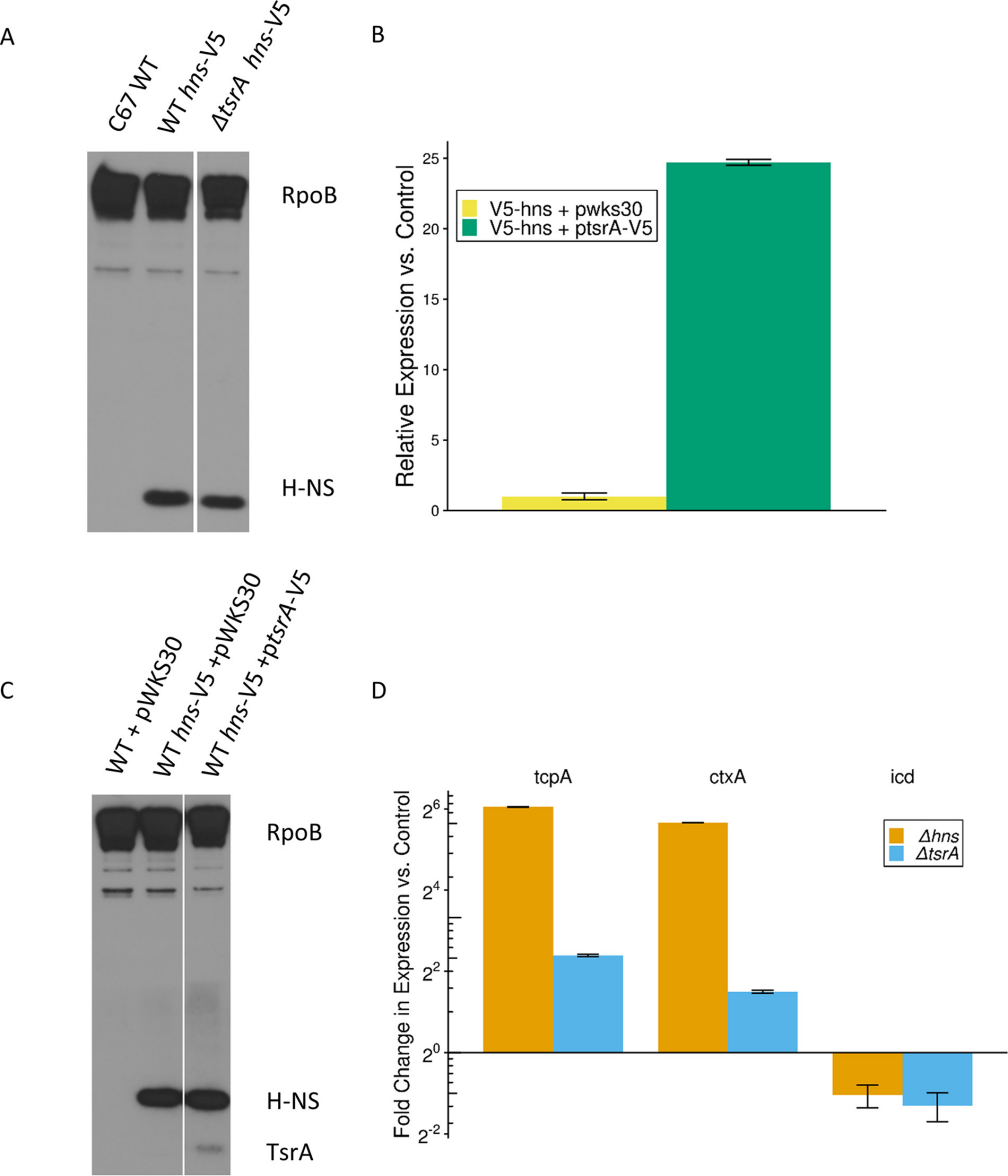
Validation of protein and RNA levels. (A) Western blot showing protein levels of H-NS across conditions. H-NS-V5 was identified using an anti-V5 antibody. An anti-RNA polymerase β subunit (RpoB) immunoblot served as the loading control. The image is representative of three biological replicates per condition. (B) Relative expression levels of *tsrA* as determined via qPCR. Expression levels normalized to 16S RNA levels under each condition. The change in expression is compared to *tsrA* expression in C6706 V5-hns strain containing the empty plasmid pWSK30. Bars indicate the standard errors of the mean for three biological replicates per condition. (C) Western blot showing relative protein levels of TsrA compared to H-NS. H-NS-V5 and TsrA-V5 were identified using an anti-V5 antibody. An anti-RNA polymerase β subunit (RpoB) immunoblot served as the loading control. Image is representative of three biological replicates per condition. (D) Relative expression levels of select genes, as determined via qPCR. Expression levels were normalized to the 16S RNA levels under each condition. The change in expression was normalized to relative levels in the C6706 strain. Bars indicate standard errors of the mean for three biological replicates per condition.

Previous work showed that TsrA regulates V. cholerae gene expression of *ctxA* and *toxT* and that TsrA is structurally similar to the oligomerization domain of H-NS (34). These observations suggested TsrA might have a similar function to H-NS. To investigate this hypothesis, we compared the global transcriptome profiles of the parental C6706 V. cholerae strain to isogenic *Δhns* and *ΔtsrA* strains (see Table S1 in the supplemental material). All strains were grown exponentially at 37°C in Luria-Bertani (LB) medium, since H-NS is known to repress virulence-associated genes under this growth condition (21). As an initial control, we verified that any effects observed upon *tsrA* deletion are not a function of decreased H-NS protein concentration by Western blotting. Using RpoB as a loading control, we saw no difference in H-NS protein levels between an H-NS-V5 strain and a *tsrA* mutant derivative of said strain, as detected with anti-V5 antibody (Fig. 1A).

10.1128/mSphere.01014-20.1 TABLE S1 Aggregate log fold change data for RNA-seq experiments. Download Table S1, XLSX file, 0.3 MB. Copyright © 2020 DuPai et al. 2020 DuPai et al. This content is distributed under the terms of the Creative Commons Attribution 4.0 International license.

**FIG 2:**
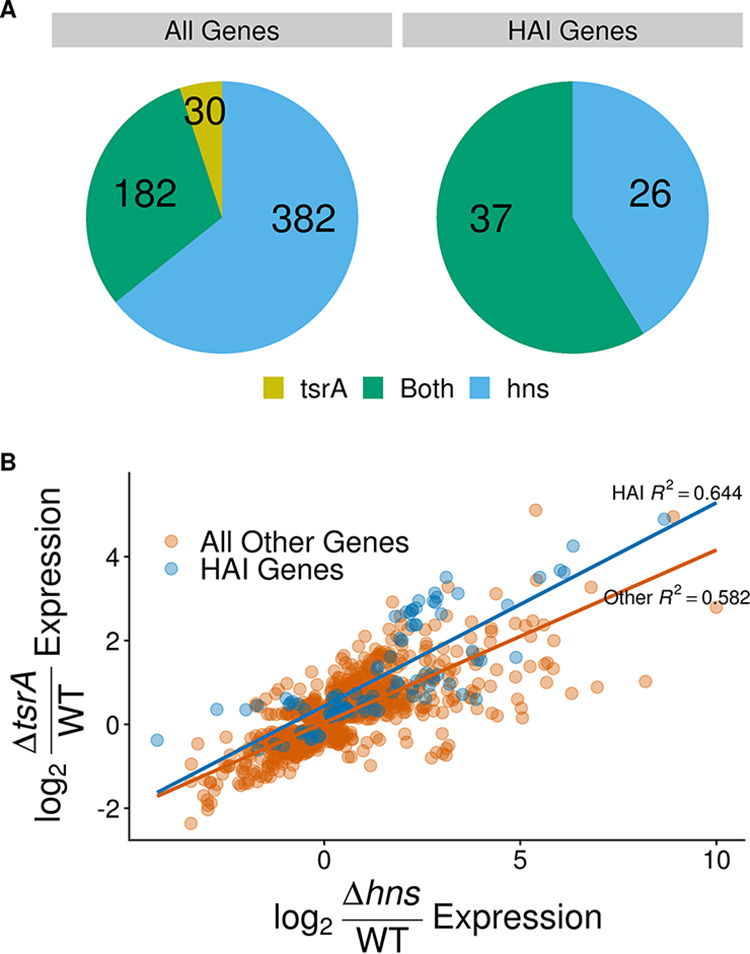
*tsrA* deletion impacts expression of H-NS regulon genes. (A) Distribution of differentially regulated genes in strains *ΔtsrA* and *Δhns* mutant strains. Blue and yellow represent the proportions of genes that are significantly differentially expressed in only the *ΔtsrA* or only the *Δhns* strain, respectively, whereas green indicates genes that are significantly differentially expressed in both strains. HAI genes include genes on VPI-1, VPI-2, VSP-1, VSP-2, and the CTX prophage. The included genes have a false discovery rate (FDR) adjusted *P* value of <0.05 and show a log_2_-fold increase or decrease in expression compared to the wild type of at least 1 in the indicated strain(s). (B) Summary of genes that are significantly differentially expressed in either of two mutant strains (*ΔtsrA* and *Δhns*). Each point represents a distinct gene. HAI genes (blue) include genes on VPI-1, VPI-2, VSP-1, VSP-2, and the CTX prophage, while “All Other Genes” (orange) include all other V. cholerae genes. Included genes have an FDR adjusted *P* value of <0.05 in both strains.

In line with previous estimates (24), our data show that the H-NS regulon encompasses nearly 600 genes (Fig. 2A; see also Table S1 in the supplemental material). These include, as expected, genes associated with virulence and T6SS (Table 1). Although generally less extreme, RNA expression changes upon deletion of *tsrA* heavily mirror those observed in the *Δhns* mutant for a large subset of genes, especially genes on HAIs (Table 1; see also Table S1). When looking at all significantly differentially expressed loci in both strains regardless of fold change, the expression levels of HAI genes were more strongly correlated (adjusted *R*^2^ = 0.644) than their progenitor genome counterparts (adjusted *R*^2^ = 0.582) (Fig. 2B). With regard to effect size across all genes that significantly changed expression by 2-fold or more in the *ΔtsrA* strain versus the wild type, 181 loci (roughly 86%) exhibited similar behavior in the *Δhns* strain (Fig. 2A). These 181 overlapping loci include all 37 HAI genes that are differentially expressed in the *ΔtsrA* strain.

**TABLE 1:** Select differentially expressed genes^*a*^

Locus tag	Gene	Δ*tsrA*		Δ*hns*		Normalized GC
		L2FC	*q* value	L2FC	*q* value	
VC0070	*tsrA*	–11.71	9.00E–21	–0.51	6.70E–01	0.98
VC1130	*hns*	–0.16	1.00E+00	–11.48	0.00E+00	0.91
						
HAI genes						
VC0184		1.75	1.30E–190	3.78	0.00E+00	0.83
VC0185		1.04	4.00E–60	2.36	0.00E+00	0.77
VC0828	*tcpA*	2.95	5.90E–285	2.41	4.10E–191	0.91
VC0829	*tcpB*	2.68	0.00E+00	2.09	2.70E–260	0.85
VC0830	*tcpQ*	2.65	1.30E–187	2.18	1.70E–128	0.75
VC0835	*tcpT*	3.03	9.50E–305	2.88	2.10E–274	0.76
VC0836	*tcpE*	3.13	0.00E+00	2.82	0.00E+00	0.75
VC0837	*tcpF*	3.51	0.00E+00	3.11	3.50E–248	0.73
VC0838	*toxT*	1.32	1.30E–69	1.34	7.50E–73	0.59
VC0841	*acfC*	2.35	1.80E–212	2.23	2.30E–191	0.76
VC0844	*acfA*	2.63	2.10E–149	2.99	1.20E–192	0.66
VC0845	*acfD*	3.12	0.00E+00	3.41	0.00E+00	0.81
VC1456	*ctxB*	2.38	2.70E–107	2.31	7.10E–102	0.68
VC1457	*ctxA*	2.37	4.10E–133	2.35	2.40E–131	0.81
VC1806		1.60	2.90E–78	4.89	0.00E+00	0.91
VC1807		4.90	3.90E–65	8.67	4.90E–209	0.68
						
T6SS genes						
VCA0105		1.86	1.90E–48	2.61	7.70E–97	1.17
VCA0106		1.93	3.50E–92	2.61	2.30E–170	0.98
VCA0107	*vipA*	2.07	2.00E–67	3.74	1.90E–234	1.02
VCA0108	*vipB*	1.52	2.80E–50	3.05	1.20E–209	1.01
						
TCA cycle						
VC0792	*oadB*	–1.70	2.60E–39	–3.08	1.30E–119	1.10
VC0793	*oadA*	–1.38	2.90E–41	–2.90	3.60E–169	1.10
VC0794	*oadG-2*	–1.32	2.30E–28	–3.06	2.90E–114	1.01
VC0800		–1.43	2.00E–42	–2.74	5.90E–147	1.13
VC0801	*citG*	–1.57	9.80E–42	–2.80	1.20E–127	1.09
						
Chitin utilization						
VC0616		1.64	3.20E–29	0.50	1.10E–03	1.03
VC0617		2.10	3.40E–55	1.04	3.30E–14	1.02
VC0618		2.92	3.80E–62	1.74	1.40E–22	1.08
VC0619		1.86	1.20E–24	0.88	2.40E–06	1.01

a The indicated genes showed significant differences in expression between one or both mutant strains and a wild-type C6706 Vibrio cholerae strain. *ΔtsrA* L2FC = log_2_(*ΔtsrA* gene abundance/wild-type gene abundance); *Δhns* L2FC = log_2_(*Δhns* gene abundance/wild-type gene abundance); *q* value = FDR adjusted *P* value; normalized GC = GC content/average chromosomal GC content.

TsrA demonstrates GC and HAI independent effects on both V. cholerae chromosomes as well (Table 1). Expression of genes associated with T6SS, such as *vipAB* (35, 36), was increased in both mutants despite exhibiting GC content comparable to background levels. These findings agree with and expand upon previously observed links between TsrA and HCP levels (34). In addition, tricarboxylic acid (TCA) cycle enzyme genes, such as *oadB* and *citG*, are downregulated in both knockout strains. Since TCA cycle products are known to repress ToxT expression in V. cholerae (37), transcriptional regulation of these genes by TsrA provides a link between cellular response to environmental cues and regulation of virulence genes. A few metabolism-related genes also appeared to be regulated by TsrA but not H-NS, most notably loci involved in chitin utilization (VC0616-VC0619) (38–40) (Table 1). In sum, TsrA, like H-NS, functions in and regulates key pathways controlling the broader V. cholerae life cycle (19).

### TsrA plays a critical role in mouse intestinal colonization.

**FIG 3:**
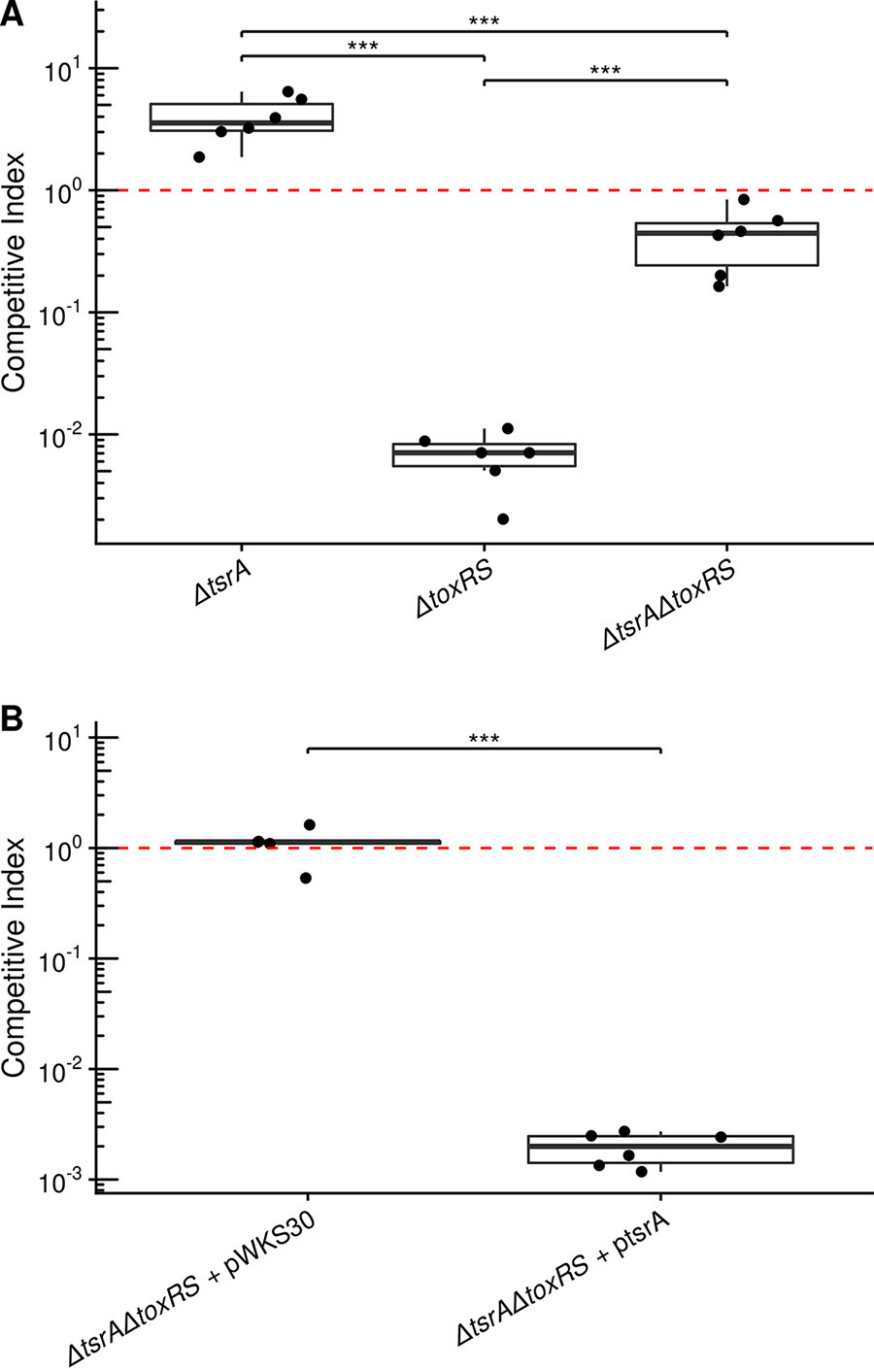
Deletion of *tsrA* promotes mouse intestinal colonization. Competitive indices of V. cholerae mutants compared to the wild type. (A and B) The indicated strains were competed with wild-type (C6706 with or without pWKS30) V. cholerae in an infant mouse infection model. The fold change of the mutant versus the wild-type strain is shown. The red line indicates perfect competition with the wild type (i.e., a competitive index of 1). Points indicate individual animals, and boxplots indicate the median, first and third quartiles, and range (***, *P* < 0.001).

Deletion of *tsrA* has been shown to increase V. cholerae colonization in an infant mouse model and affect expression of a few genes dually regulated by H-NS and ToxR (34). We previously showed the importance of ToxR in V. cholerae host colonization could be abrogated by deleting H-NS (12). Given the ability of TsrA to regulate virulence gene expression and its parallel effects with H-NS, we hypothesized that TsrA may likewise play a critical role in host colonization. We used an infant mouse model of intestinal colonization to test this hypothesis. As found previously (34), deletion of *tsrA* leads to a modest hypercolonization of the infant mouse with the *ΔtsrA* mutant out-colonizing a wild-type C6706 strain by ∼4-fold (Fig. 3). Remarkably, we show that deletion of *tsrA* completely negates V. cholerae’s dependence on ToxR to colonize the infant mouse intestine (Fig. 3A). The near wild-type infection levels of the *ΔtsrA ΔtoxRS* strain are in stark contrast to the drastically reduced infectivity seen in the *ΔtoxRS* single mutant, providing a clear testament to the potency of TsrA-mediated virulence repression. This phenotype was complemented by exogenous expression of *tsrA* in the *ΔtsrA ΔtoxRS* mutant, which showed an extreme colonization defect in line with the *ΔtoxRS* single mutant (Fig. 3B). These results implicate TsrA as a high level regulator of critical V. cholerae virulence systems.

## DISCUSSION

Our results support previous TsrA research and suggest an expanded role for this protein in fine-tuning expression of the complex virulence cascade of V. cholerae. As a testament to TsrA’s importance, deletion of *tsrA* wholly overcomes the infant mouse intestinal colonization defects seen in a *ΔtoxRS* strain. We further show that TsrA stands as a potent coregulator of HAI genes and other portions of the H-NS regulon most responsible for virulence. TsrA’s regulatory activities mirror and supplement those of H-NS.

Since virulent strains of V. cholerae rely on ToxR and its regulon to facilitate intestinal colonization (4, 41), it is little surprise that H-NS and TsrA regulons overlap so heavily at sites, such as VPI-1 and the CTX prophage, that are also controlled by ToxR (21, 23). TsrA-mediated repression at these loci likely explains how a *ΔtsrA ΔtoxRS* strain maintains wild-type levels of intestinal colonization, a phenotype previously observed in a *Δhns ΔtoxRS* mutant (12).

Since TsrA lacks a DNA binding domain but has some weak homology to the H-NS oligomerization domain (34), it may act through interactions with H-NS that affect the latter’s ability to influence gene expression, as HHA is known to do in Escherichia coli (28, 42, 43). Unfortunately, we were unable to purify TsrA after multiple attempts to confirm an interaction with H-NS *in vitro*. It is clear that future genetic and biochemical studies will be needed to fully determine how TsrA functions and influences H-NS activity.

TsrA’s low relative protein abundance compared to H-NS clarifies the smaller effect size of most transcriptomic changes in the *ΔtsrA* strain compared to more intense changes in the *Δhns* strain. These data, as well as TsrA’s known role in coordinating T6SS expression in coordination with quorum-sensing pathways, generally support a model of TsrA having a more specialized function than H-NS. In this model, if H-NS is the master regulator of virulence gene expression, then TsrA is a master modulator, fine-tuning expression levels in response to some unknown environmental cues.

Our results suggest that TsrA serves an important role in V. cholerae gene regulation by controlling the expression of key virulence genes and other H-NS targets. Since V. cholerae’s survival in diverse environments depends on precise control of varied genomic elements at specific times, a factor such as TsrA that can modulate and target expression of specific genes helps potentiate V. cholerae’s impressive proclivity to adapt and thrive.

## MATERIALS AND METHODS

### Bacterial strains, plasmids, and media.

Strains and plasmids used in this study are listed in Table S1 in the supplemental material. Strains were grown in lysogeny broth/agar at 37°C. The antibiotics streptomycin (100 μg/ml) and carbenicillin (75 μg/ml) were used for selection as needed. X-Gal (5-bromo-4-chloro-3-indolyl-β-d-galactopyranoside) was used at 40 μg/ml.

### Plasmid and strain construction.

All cloning products were sequence verified. For in-frame deletion constructs, surrounding genomic DNA was amplified by crossover PCR and cloned into pWM91 for subsequent *sacB*-mediated allelic exchange (44). To add the V5 epitope tag to H-NS, *hns* was amplified from the genome using primers, including the epitope sequences, to add the appropriate tag to the resulting PCR product. For complementation constructs, the original genes with their native promoters were PCR amplified off chromosomal DNA and cloned into plasmid pWKS30 (45).

### Western blot analysis.

Equal amounts of cells grown at 37°C in LB medium were harvested during exponential phase. Cells were pelleted, resuspended in loading buffer, and separated on a NuPAGE Bis-Tris gel (Thermo Fisher). After transfer, membranes were blotted with monoclonal anti-V5 antibody (Sigma-Aldrich) or anti-RpoB antibody (BioLegend). RpoB was blotted as a loading control. Pierce ECL Western blotting substrate (Thermo Scientific) was added before exposing the X-ray film. Experiments were carried out in at least biological triplicates.

### RNA sequencing.

RNA sequencing (RNA-seq) was performed essentially as previously described (46). Total RNA was extracted from cells in exponential phase growing at 37°C in LB medium using a Direct-zol RNA MiniPrep kit with TRI-Reagent (Zymo Research). DNase treatment was carried out using a Turbo DNA-free kit (Ambion, Inc.). Ribosomal RNA was depleted using a Ribo-Zero rRNA removal kit for Gram-negative bacteria (Illumina). Sequencing libraries were then prepared for the Illumina sequencing platform. Experiments were repeated in biological triplicates.

### Data analysis and visualization.

RNA-seq data were aligned to a transcriptome (47) derived from the El Tor N16961 reference genome (ASM674v1) (48). RNA abundances were quantified using Kallisto version 0.43.1 (49), and differential expression was calculated using DESeq2 version 1.18.1 (50). All other data were analyzed using R version 3.6 (51) with the Tidyverse family of packages (52). All visualizations were generated with ggplot2 version 3.2.1 (53).

### Infant mouse colonization assays.

Assays were performed as previously described (12). At least five mice were tested for each mutant. *P* values were calculated using Tukey’s honest significant difference test following one-way analysis of variance.

### Ethics approval.

The mouse experiment was reviewed and approved by the UT Austin IACUC (approval AUP-2018-00354).

### Data availability.

Raw sequence reads for the RNA-seq data were uploaded to the Sequence Read Archive (SRA) (https://www.ncbi.nlm.nih.gov/sra) under accession number SRP242320. Processed RNA-seq results are provided in Table S1 in the supplemental material.

10.1128/mSphere.01014-20.2 TABLE S2 Strains and plasmids used in this study. Download Table S2, XLSX file, 0.01 MB. Copyright © 2020 DuPai et al. 2020 DuPai et al. This content is distributed under the terms of the Creative Commons Attribution 4.0 International license.
